# Isopropyl­aminium 2-carb­oxy-4,5-di­chloro­benzoate

**DOI:** 10.1107/S1600536809052672

**Published:** 2009-12-12

**Authors:** Graham Smith, Urs D. Wermuth

**Affiliations:** a School of Physical and Chemical Sciences, Queensland University of Technology, GPO Box 2434, Brisbane, Queensland 4001, Australia; b School of Biomolecular and Physical Sciences, Griffith University, Nathan, Queensland 4111, Australia

## Abstract

In the structure of the 1:1 proton-transfer compound of isopropyl­amine with 4,5-dichloro­phthalic acid, C_3_H_10_N^+^·C_8_H_3_Cl_2_O_4_^−^, the three cation H-atom donors associate with three separate carboxyl O-atom anion acceptors, giving conjoint cyclic *R*_4_^4^(12), *R*_4_^4^(16) hydrogen-bonding cation–anion inter­actions in a one-dimensional ribbon structure. In the anions, the carboxyl groups lie slightly out of the plane of the benzene ring [maximum deviations = 0.439 (1) for a carboxylic acid O atom and 0.433 (1) Å for a carboxyl­ate O atom]. However, the *syn*-related proton of the carboxylic acid group forms the common short intra­molecular O—H⋯O_carbox­yl_ hydrogen bond.

## Related literature

For the structures of other hydrogen 4,5-dichloro­phthalate salts, see: Mattes & Dorau (1986 ▶); Mallinson *et al.* (2003 ▶); Bozkurt *et al.* (2006 ▶); Odabaşoğlu & Büyükgüngör (2007 ▶); Smith *et al.* (2007 ▶, 2008*a* ▶,*b* ▶, 2009*a* ▶,*b* ▶,*c* ▶); Smith & Wermuth (2010 ▶). For graph-set analysis see: Etter *et al.* (1990 ▶).
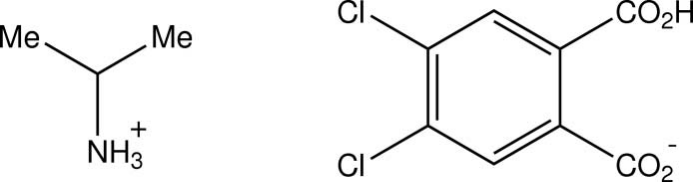


## Experimental

### 

#### Crystal data

C_3_H_10_N^+^·C_8_H_3_Cl_2_O_4_^−^
*M* *_r_* = 294.12
Monoclinic, 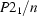

*a* = 5.8362 (7) Å
*b* = 21.040 (2) Å
*c* = 10.3641 (13) Å
β = 95.064 (12)°
*V* = 1267.7 (3) Å^3^
*Z* = 4
Mo *K*α radiation
μ = 0.52 mm^−1^
*T* = 200 K
0.40 × 0.20 × 0.18 mm

#### Data collection

Oxford Diffraction Gemini-S CCD detector diffractometer
Absorption correction: multi-scan (*SADABS*; Sheldrick, 1996 ▶) *T*_min_ = 0.942, *T*_max_ = 0.982
8508 measured reflections
2484 independent reflections
2103 reflections with *I* > 2σ(*I*)
*R*_int_ = 0.020

#### Refinement

*R*[*F* ^2^ > 2σ(*F* ^2^)] = 0.027
*wR*(*F* ^2^) = 0.070
*S* = 1.11
2484 reflections
179 parameters
H atoms treated by a mixture of independent and constrained refinement
Δρ_max_ = 0.24 e Å^−3^
Δρ_min_ = −0.22 e Å^−3^

### 

Data collection: *CrysAlis PRO* (Oxford Diffraction, 2009 ▶); cell refinement: *CrysAlis PRO*; data reduction: *CrysAlis PRO*; program(s) used to solve structure: *SIR92* (Altomare *et al.*, 1994 ▶); program(s) used to refine structure: *SHELXL97* (Sheldrick, 2008 ▶) within *WinGX* (Farrugia, 1999 ▶); molecular graphics: *PLATON* (Spek, 2009 ▶); software used to prepare material for publication: *PLATON*.

## Supplementary Material

Crystal structure: contains datablocks global, I. DOI: 10.1107/S1600536809052672/sj2696sup1.cif

Structure factors: contains datablocks I. DOI: 10.1107/S1600536809052672/sj2696Isup2.hkl

Additional supplementary materials:  crystallographic information; 3D view; checkCIF report

## Figures and Tables

**Table 1:** Hydrogen-bond geometry (Å, °)

*D*—H⋯*A*	*D*—H	H⋯*A*	*D*⋯*A*	*D*—H⋯*A*
O12—H12⋯O21	1.00 (3)	1.45 (3)	2.4507 (16)	179 (3)
N1*A*—H11*A*⋯O11	0.977 (18)	1.875 (18)	2.8175 (17)	161.2 (15)
N1*A*—H12*A*⋯O21^i^	0.876 (19)	2.021 (18)	2.8593 (16)	159.6 (16)
N1*A*—H13*A*⋯O22^ii^	0.92 (2)	1.98 (2)	2.8869 (17)	168.8 (15)

Symmetry codes: (i) 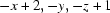; (ii) 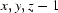.

**Fig. 1.**
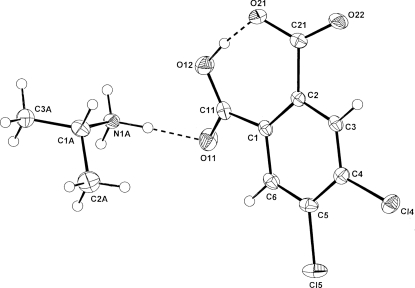
Molecular configuration and atom numbering scheme for the isopropylaminium cation and the hydrogen 4,5-dichlorophthalate anion in (I). Non-H atoms are shown as 40% probability displacement ellipsoids. The inter-species hydrogen bond is shown as a dashed line.

**Fig. 2.**
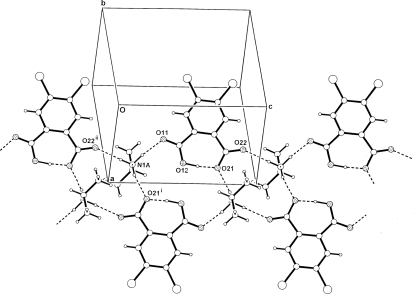
The one-dimensional hydrogen-bonded ribbon structure of (I) extending across the c axial direction in the unit cell. Hydrogen bonds are shown as dashed lines. For symmetry codes see Table 1.

## supplementary crystallographic information

### Comment

The 1:1 proton-transfer compounds of 4,5-dichlorophthalic acid (DCPA) with a number of nitrogen Lewis bases commonly have low-dimensional hydrogen-bonded structures (Smith et al., 2007, 2008a,b, 2009a,b,c; Smith & Wermuth, 2010). In the majority of these structures, the DCPA anions are essentially planar with short intramolecular carboxylic acid O–H···O_carboxyl_ hydrogen bonds. These features were therefore expected and found in the 1:1 proton-transfer compound of DCPA with isopropylamine, the title compound C_3_H_10_N^+^ C_8_H_3_Cl_2_O_4_^-^ (I), reported here.

In (I), the aminium group of the cation forms N^+^–H···O_carboxyl_ hydrogen bonds with O acceptors of three separate DCPA anions (Figs. 1, 2). These associations (Table 1) give one-dimensional ribbon structures which extend across the c cell direction in the unit cell (Fig. 2) and feature conjoint cyclic R_4_^4^(12) and R_4_^4^(16) cation–anion hydrogen-bonding interactions (Etter et al., 1990). Within the DCPA anion [torsion angles C2–C1–C11–O11, -161.01 (13)° and C1–C2–C21–O22, -156.69 (13)°] indicate greater distortion from planarity than has been found in the common `planar' DCPA anion examples. The short intramolecular O–H···O_carboxyl_ hydrogen bond is also slightly longer [2.4507 (16) Å] (cf. 1.4054 (19) Å (Smith et al., 2009c). Associated with this bond is a significant distortion of the exo-C1 and C2 bond angles [C1–C2–C21, 128.14 (11) ° and C2–C1–C11, 128.32 (11) °]. This and a lengthening of the C1–C11 and C2–C21 bonds [1.5189 (18) and 1.5297 (18) Å], as well as short intramolecular aromatic ring C–H···O_carboxyl_ interactions [2.6853 (18), 2.6996 (17) Å], are features of the `planar' hydrogen DCPA anions which have been noted previously (Smith et al., 2009c).

### Experimental

The title compound (I) was synthesized by heating together 1 mmol quantities of isopropylamine and 4,5-dichlorophthalic acid in 50 ml of methanol for 10 min under reflux. After concentration to *ca*. 30 ml, total room-temperature evaporation of the hot-filtered solution gave a white non-crystalline solid which was redissolved in water, finally providing colourless flat prisms (m.p. 533 K).

### Refinement

Hydrogen atoms potentially involved in hydrogen-bonding interactions were located by difference methods and their positional and isotropic displacement parameters were refined. Other H atoms were included in the refinement at calculated positions [C–H_aromatic_ = 0.93 Å; C–H_aliphatic_ = 0.96–0.98 Å] and treated as riding models with *U*_iso_(H) = 1.2*U*_eq_ (C).

### Crystal data

**Table:**

C_3_H_10_N^+^·C_8_H_3_Cl_2_O_4_^−^	*F*(000) = 608
*M**_r_* = 294.12	*D*_x_ = 1.541 Mg m^−^^3^
Monoclinic, *P*2_1_/*n*	Melting point: 533 K
Hall symbol: -P 2yn	Mo *K*α radiation, λ = 0.71073 Å
*a* = 5.8362 (7) Å	Cell parameters from 3787 reflections
*b* = 21.040 (2) Å	θ = 3.5–28.9°
*c* = 10.3641 (13) Å	µ = 0.52 mm^−^^1^
β = 95.064 (12)°	*T* = 200 K
*V* = 1267.7 (3) Å^3^	Prism, colourless
*Z* = 4	0.40 × 0.20 × 0.18 mm

### Data collection

**Table:**

Oxford Diffraction Gemini-S CCD detector diffractometer	2484 independent reflections
Radiation source: Enhance (Mo) X-ray source	2103 reflections with *I* > 2σ(*I*)
graphite	*R*_int_ = 0.020
ω scans	θ_max_ = 26.0°, θ_min_ = 3.5°
Absorption correction: multi-scan (*SADABS*; Sheldrick, 1996)	*h* = −7→7
*T*_min_ = 0.942, *T*_max_ = 0.982	*k* = −25→25
8508 measured reflections	*l* = −11→12

### Refinement

**Table:**

Refinement on *F*^2^	Primary atom site location: structure-invariant direct methods
Least-squares matrix: full	Secondary atom site location: difference Fourier map
*R*[*F*^2^ > 2σ(*F*^2^)] = 0.027	Hydrogen site location: inferred from neighbouring sites
*wR*(*F*^2^) = 0.070	H atoms treated by a mixture of independent and constrained refinement
*S* = 1.11	*w* = 1/[σ^2^(*F*_o_^2^) + (0.0418*P*)^2^] where *P* = (*F*_o_^2^ + 2*F*_c_^2^)/3
2484 reflections	(Δ/σ)_max_ = 0.001
179 parameters	Δρ_max_ = 0.24 e Å^−^^3^
0 restraints	Δρ_min_ = −0.22 e Å^−^^3^

### Special details

**Table:**

Geometry. Bond distances, angles *etc*. have been calculated using the rounded fractional coordinates. All su's are estimated from the variances of the (full) variance-covariance matrix. The cell e.s.d.'s are taken into account in the estimation of distances, angles and torsion angles
Refinement. Refinement of *F*^2^ against ALL reflections. The weighted *R*-factor *wR* and goodness of fit *S* are based on *F*^2^, conventional *R*-factors *R* are based on *F*, with *F* set to zero for negative *F*^2^. The threshold expression of *F*^2^ > σ(*F*^2^) is used only for calculating *R*-factors(gt) *etc*. and is not relevant to the choice of reflections for refinement. *R*-factors based on *F*^2^ are statistically about twice as large as those based on *F*, and *R*- factors based on ALL data will be even larger.

### Fractional atomic coordinates and isotropic or equivalent isotropic displacement parameters (Å^2^)

**Table:**

	*x*	*y*	*z*	*U*_iso_*/*U*_eq_	
Cl4	−0.02700 (6)	0.21328 (2)	0.79371 (4)	0.0321 (1)	
Cl5	−0.07644 (7)	0.22223 (2)	0.48482 (4)	0.0381 (1)	
O11	0.51763 (18)	0.06697 (6)	0.34762 (10)	0.0380 (4)	
O12	0.79781 (18)	0.04757 (5)	0.49757 (11)	0.0363 (4)	
O21	0.85388 (16)	0.04986 (5)	0.73467 (10)	0.0284 (3)	
O22	0.61961 (18)	0.05281 (5)	0.89173 (10)	0.0319 (3)	
C1	0.4642 (2)	0.10656 (6)	0.55707 (13)	0.0208 (4)	
C2	0.4887 (2)	0.10301 (6)	0.69407 (13)	0.0197 (4)	
C3	0.3362 (2)	0.13730 (6)	0.76294 (14)	0.0214 (4)	
C4	0.1622 (2)	0.17415 (6)	0.70180 (14)	0.0230 (4)	
C5	0.1406 (2)	0.17807 (6)	0.56719 (14)	0.0247 (4)	
C6	0.2902 (2)	0.14478 (6)	0.49726 (14)	0.0241 (4)	
C11	0.5991 (2)	0.07119 (6)	0.46084 (14)	0.0250 (4)	
C21	0.6660 (2)	0.06527 (6)	0.78049 (14)	0.0225 (4)	
N1A	0.8171 (2)	0.04013 (6)	0.15582 (13)	0.0231 (4)	
C1A	0.9564 (2)	0.10018 (7)	0.17227 (15)	0.0271 (4)	
C2A	0.7999 (3)	0.15688 (8)	0.14539 (19)	0.0420 (6)	
C3A	1.1460 (3)	0.09810 (8)	0.08332 (17)	0.0350 (5)	
H3	0.35150	0.13540	0.85290	0.0260*	
H6	0.27510	0.14780	0.40740	0.0290*	
H12	0.821 (4)	0.0490 (11)	0.594 (3)	0.087 (8)*	
H1A	1.02480	0.10260	0.26190	0.0330*	
H11A	0.694 (3)	0.0418 (8)	0.2138 (18)	0.040 (5)*	
H12A	0.907 (3)	0.0072 (9)	0.1720 (17)	0.036 (5)*	
H13A	0.751 (3)	0.0384 (8)	0.072 (2)	0.037 (5)*	
H21A	0.68170	0.15660	0.20420	0.0500*	
H22A	0.73080	0.15470	0.05790	0.0500*	
H23A	0.88790	0.19530	0.15700	0.0500*	
H31A	1.24140	0.06160	0.10360	0.0420*	
H32A	1.23720	0.13600	0.09430	0.0420*	
H33A	1.08070	0.09530	−0.00480	0.0420*	

### Atomic displacement parameters (Å^2^)

**Table:**

	*U*^11^	*U*^22^	*U*^33^	*U*^12^	*U*^13^	*U*^23^
Cl4	0.0289 (2)	0.0304 (2)	0.0377 (2)	0.0045 (1)	0.0069 (2)	−0.0040 (2)
Cl5	0.0363 (2)	0.0352 (2)	0.0399 (3)	0.0086 (2)	−0.0133 (2)	0.0045 (2)
O11	0.0356 (6)	0.0582 (7)	0.0205 (6)	−0.0017 (5)	0.0050 (5)	−0.0080 (5)
O12	0.0341 (6)	0.0470 (6)	0.0286 (7)	0.0119 (5)	0.0069 (5)	0.0007 (5)
O21	0.0221 (5)	0.0317 (6)	0.0309 (6)	0.0035 (4)	−0.0002 (4)	0.0045 (4)
O22	0.0368 (6)	0.0395 (6)	0.0187 (6)	0.0064 (4)	−0.0014 (4)	0.0048 (4)
C1	0.0216 (7)	0.0217 (7)	0.0189 (7)	−0.0057 (5)	0.0008 (5)	0.0006 (5)
C2	0.0196 (6)	0.0194 (6)	0.0196 (7)	−0.0048 (5)	−0.0010 (5)	0.0012 (5)
C3	0.0248 (7)	0.0226 (7)	0.0168 (7)	−0.0038 (5)	0.0011 (5)	0.0007 (5)
C4	0.0223 (7)	0.0200 (7)	0.0268 (8)	−0.0029 (5)	0.0024 (6)	−0.0014 (5)
C5	0.0249 (7)	0.0208 (7)	0.0268 (8)	−0.0018 (5)	−0.0069 (6)	0.0028 (5)
C6	0.0275 (7)	0.0265 (7)	0.0174 (8)	−0.0055 (5)	−0.0033 (6)	0.0021 (5)
C11	0.0273 (7)	0.0268 (7)	0.0214 (8)	−0.0060 (6)	0.0052 (6)	0.0006 (6)
C21	0.0246 (7)	0.0208 (7)	0.0212 (8)	−0.0029 (5)	−0.0030 (6)	−0.0004 (5)
N1A	0.0237 (6)	0.0269 (6)	0.0183 (7)	0.0018 (5)	−0.0003 (5)	0.0007 (5)
C1A	0.0315 (8)	0.0281 (7)	0.0208 (8)	−0.0026 (6)	−0.0031 (6)	−0.0018 (6)
C2A	0.0512 (10)	0.0287 (8)	0.0474 (11)	0.0071 (7)	0.0111 (8)	−0.0027 (7)
C3A	0.0291 (8)	0.0354 (8)	0.0408 (10)	−0.0019 (6)	0.0041 (7)	0.0071 (7)

### Geometric parameters (Å, °)

**Table:**

Cl4—C4	1.7292 (14)	C2—C3	1.3908 (18)
Cl5—C5	1.7337 (14)	C3—C4	1.3858 (18)
O11—C11	1.2298 (18)	C4—C5	1.392 (2)
O12—C11	1.2880 (16)	C5—C6	1.3747 (18)
O21—C21	1.2747 (16)	C3—H3	0.9300
O22—C21	1.2354 (18)	C6—H6	0.9300
O12—H12	1.00 (3)	C1A—C2A	1.513 (2)
N1A—C1A	1.5039 (19)	C1A—C3A	1.502 (2)
N1A—H11A	0.977 (18)	C1A—H1A	0.9800
N1A—H13A	0.92 (2)	C2A—H21A	0.9600
N1A—H12A	0.876 (19)	C2A—H22A	0.9600
C1—C6	1.3968 (18)	C2A—H23A	0.9600
C1—C11	1.5189 (18)	C3A—H31A	0.9600
C1—C2	1.4164 (19)	C3A—H32A	0.9600
C2—C21	1.5297 (18)	C3A—H33A	0.9600
			
C11—O12—H12	108.9 (14)	O21—C21—C2	118.27 (12)
H11A—N1A—H12A	111.8 (15)	O22—C21—C2	117.64 (11)
C1A—N1A—H13A	108.5 (11)	C2—C3—H3	119.00
C1A—N1A—H11A	108.6 (10)	C4—C3—H3	119.00
H11A—N1A—H13A	108.2 (16)	C1—C6—H6	119.00
H12A—N1A—H13A	110.1 (16)	C5—C6—H6	119.00
C1A—N1A—H12A	109.5 (12)	C2A—C1A—C3A	112.05 (14)
C6—C1—C11	112.92 (12)	N1A—C1A—C2A	109.27 (11)
C2—C1—C6	118.71 (12)	N1A—C1A—C3A	109.03 (12)
C2—C1—C11	128.32 (11)	N1A—C1A—H1A	109.00
C1—C2—C21	128.14 (11)	C2A—C1A—H1A	109.00
C1—C2—C3	118.33 (12)	C3A—C1A—H1A	109.00
C3—C2—C21	113.53 (12)	C1A—C2A—H21A	110.00
C2—C3—C4	122.13 (13)	C1A—C2A—H22A	109.00
Cl4—C4—C5	121.21 (10)	C1A—C2A—H23A	109.00
C3—C4—C5	119.29 (12)	H21A—C2A—H22A	109.00
Cl4—C4—C3	119.49 (11)	H21A—C2A—H23A	109.00
Cl5—C5—C4	121.52 (10)	H22A—C2A—H23A	110.00
C4—C5—C6	119.51 (12)	C1A—C3A—H31A	109.00
Cl5—C5—C6	118.94 (11)	C1A—C3A—H32A	110.00
C1—C6—C5	122.01 (13)	C1A—C3A—H33A	109.00
O11—C11—O12	121.09 (13)	H31A—C3A—H32A	109.00
O11—C11—C1	118.87 (11)	H31A—C3A—H33A	109.00
O12—C11—C1	120.03 (12)	H32A—C3A—H33A	109.00
O21—C21—O22	124.07 (12)		
			
C6—C1—C2—C3	0.72 (18)	C1—C2—C21—O21	22.83 (19)
C6—C1—C2—C21	−179.34 (12)	C1—C2—C21—O22	−158.69 (13)
C11—C1—C2—C3	−176.37 (12)	C3—C2—C21—O21	−157.23 (12)
C11—C1—C2—C21	3.6 (2)	C3—C2—C21—O22	21.26 (17)
C2—C1—C6—C5	−1.04 (19)	C2—C3—C4—Cl4	177.78 (10)
C11—C1—C6—C5	176.47 (11)	C2—C3—C4—C5	−1.10 (19)
C2—C1—C11—O11	161.01 (13)	Cl4—C4—C5—Cl5	−0.02 (16)
C2—C1—C11—O12	−20.7 (2)	Cl4—C4—C5—C6	−178.09 (10)
C6—C1—C11—O11	−16.22 (17)	C3—C4—C5—Cl5	178.86 (10)
C6—C1—C11—O12	162.10 (12)	C3—C4—C5—C6	0.78 (18)
C1—C2—C3—C4	0.34 (19)	Cl5—C5—C6—C1	−177.84 (10)
C21—C2—C3—C4	−179.61 (11)	C4—C5—C6—C1	0.29 (19)

### Hydrogen-bond geometry (Å, °)

**Table:**

*D*—H···*A*	*D*—H	H···*A*	*D*···*A*	*D*—H···*A*
O12—H12···O21	1.00 (3)	1.45 (3)	2.4507 (16)	179 (3)
N1A—H11A···O11	0.977 (18)	1.875 (18)	2.8175 (17)	161.2 (15)
N1A—H12A···O21^i^	0.876 (19)	2.021 (18)	2.8593 (16)	159.6 (16)
N1A—H13A···O22^ii^	0.92 (2)	1.98 (2)	2.8869 (17)	168.8 (15)
C3—H3···O22	0.93	2.35	2.6996 (17)	102
C6—H6···O11	0.93	2.33	2.6853 (18)	102
C3A—H31A···O22^i^	0.96	2.54	3.458 (2)	160

Symmetry codes: (i) −*x*+2, −*y*, −*z*+1; (ii) *x*, *y*, *z*−1.

## References

[bb1] Altomare, A., Cascarano, G., Giacovazzo, C., Guagliardi, A., Burla, M. C., Polidori, G. & Camalli, M. (1994). *J. Appl. Cryst.* **27**, 435.

[bb2] Bozkurt, E., Kartal, I., Odabaşoğlu, M. & Büyükgüngör, O. (2006). *Acta Cryst.* E**62**, o4258–o4260.

[bb3] Etter, M. C., MacDonald, J. C. & Bernstein, J. (1990). *Acta Cryst.* B**46**, 256–262. 10.1107/s0108768189012929 2344397

[bb4] Farrugia, L. J. (1999). *J. Appl. Cryst.* **32**, 837–838.

[bb5] Mallinson, P. R., Smith, G. T., Wilson, C. C., Grech, E. & Wozniak, K. (2003). *J. Am. Chem. Soc.* **125**, 4259–4270. 10.1021/ja029389b 12670248

[bb6] Mattes, R. & Dorau, A. (1986). *Z. Naturforsch. Teil B*, **41**, 808–814.

[bb7] Odabaşoğlu, M. & Büyükgüngör, O. (2007). *Acta Cryst.* E**63**, o4374–o4375.

[bb8] Oxford Diffraction (2009). *CrysAlis PRO* Oxford Diffraction Ltd, Yarnton, Oxfordshire, England.

[bb9] Sheldrick, G. M. (1996). *SADABS* University of Göttingen, Germany.

[bb10] Sheldrick, G. M. (2008). *Acta Cryst.* A**64**, 112–122. 10.1107/S0108767307043930 18156677

[bb11] Smith, G. & Wermuth, U. D. (2010). *J. Chem. Crystallogr.* In the press.

[bb12] Smith, G., Wermuth, U. D. & White, J. M. (2007). *Acta Cryst.* E**63**, o4276–o4277.

[bb13] Smith, G., Wermuth, U. D. & White, J. M. (2008*a*). *Acta Cryst.* C**64**, o180–o183. 10.1107/S0108270108004034 18322349

[bb14] Smith, G., Wermuth, U. D. & White, J. M. (2008*b*). *Acta Cryst.* C**64**, o532–o536. 10.1107/S0108270108027054 18758028

[bb15] Smith, G., Wermuth, U. D. & White, J. M. (2009*a*). *Acta Cryst.* C**65**, o103–o107. 10.1107/S0108270109004405 19265221

[bb16] Smith, G., Wermuth, U. D. & White, J. M. (2009*b*). *Acta Cryst.* E**65**, o2111. 10.1107/S160053680903044X PMC2970019 21577526

[bb17] Smith, G., Wermuth, U. D. & White, J. M. (2009*c*). *Acta Cryst.* E**65**, o2333. 10.1107/S1600536809034448 PMC2970357 21577804

[bb18] Spek, A. L. (2009). *Acta Cryst.* D**65**, 148–155. 10.1107/S090744490804362X PMC2631630 19171970

